# A comparison of the therapeutic efficacy of Tenofovir Disoproxil Fumarate and Entecavir in patients with chronic Hepatitis-B

**DOI:** 10.12669/pjms.40.10.10307

**Published:** 2024-11

**Authors:** Huan Wang, Liping Wu

**Affiliations:** 1 Huan Wang Department of Infectious, First People’s Hospital of Linping District, Hangzhou, Zhejiang Province 311100, P.R. China; 2 Liping Wu Department of General Geriatrics, Linping District Integrated Traditional, Chinese and Western Medicine Hospital, Hangzhou, Zhejiang Province 311100, P.R. China

**Keywords:** Tenofovir disoproxil fumarate, Entecavir (ETV), Chronic Hepatitis-B, Hepatitis-B virus deoxyribonucleic acid, HBeAg seroconversion

## Abstract

**Objective:**

To compare the therapeutic efficacy of tenofovir disoproxil fumarate (TDF) and entecavir (ETV) in patients with chronic Hepatitis-B (CHB).

**Methods:**

This retrospective study included 110 patients with CHB who received treatment at The First People’s Hospital of Linping District, Hangzhou from January 2021 to January 2023. Clinical data of the patients were reviewed and the patients were classified according to the treatment received: TDF group (n=53, patients received TDF treatment) and ETV group (n=57, patients received ETV treatment). Hepatitis-B virus deoxyribonucleic acid (HBV DNA) levels, liver function indicators, hepatitis-B e antigen (HBeAg) seroconversion rate, alanine transaminase (ALT) normalization rate, HBV DNA negative conversion rate, overall efficacy, and incidence of adverse reactions were compared.

**Results:**

The total efficacy of the treatment in the TDF group was 94.33%, significantly higher than that in the ETV group (78.95%; *P*<0.05). After the treatment, the HBV DNA levels in both groups decreased compared to pretreatment levels, and were significantly lower in the TDF group compared to the ETV group (*P*<0.05). Both groups showed significant post-treatment improvement in liver function that was markedly better in the TDF group compared to the ETV group (*P*<0.05). The HBeAg seroconversion rate, ALT normalization rate, and HBV DNA conversion rate in the TDF group were significantly higher compared to the ETV group (*P*<0.05). There was no difference in the incidence of adverse reactions between the two groups.

**Conclusions:**

Compared with ETV, TDF has comparable adverse reaction profile but has more significant clinical effects in patients with CHB, improving HBeAg seroconversion rate, ALT normalization rate, and HBV DNA negative conversion rate. TDF is associated with lower HBV DNA levels after treatment and better improvements in liver function of patients.

## INTRODUCTION

Chronic Hepatitis-B (CHB) is a long-term chronic inflammation of the liver caused by Hepatitis-B virus (HBV) infection.^1^ It can gradually develop into a chronic disease within months or years, leading to liver tissue damage and fibrosis, and subsequently, to serious complications such as cirrhosis, liver failure, and liver cancer.^1^,^2^ WHO estimates that 254 million people were living with CHB infection in 2022, with 1.2 million new infections each year. In 2022, Hepatitis-B was estimated to cause 1.1 million deaths. Although people of any age can be infected with HBV transmitted by bodily fluids, populations such as newborns, children, young adults, and healthcare workers are all at higher risk of infection.^3^,^4^ Therefore, timely preventive measures and HBV vaccination are effective means to prevent CHB.

Antiviral therapy is the main method of routine CHB treatment, and is able to reduce liver inflammation and damage by inhibiting the replication and reproduction of the virus.^5^,^6^ The commonly used antiviral drugs include nucleotide analogues (such as lamivudine, adefovir ester) and nucleoside analogues (such as Entecavir[ETV]).^6^ ETV has the advantage of effectively inhibiting HBV, reducing liver inflammation, and thus reducing the risk of further liver damage and disease progression.^7^ However, it requires long-term use and may lead to drug resistance.^6^,^7^ Tenofovir Disoproxil Fumarate (TDF), oral prodrug for tenofovir, also used to treat HBV and human immunodeficiency virus (HIV) infections.^8^ This drug can effectively reduce the viral load, inhibit virus replication and reproduction, thereby alleviating the condition and delaying disease progression.^8^,^9^ Moreover, the resistance rate of TDF is relatively low compared to other antiviral drugs.^9^

While many studies have shown that TDF and ETV are similarly effective and safe in patients with CHB^10^,^11^, there are also studies that show TDF to be superior to ETV^12^,^13^. Therefore, this study aimed to compare the therapeutic efficacy of TDF and ETV in patients with CHB to provide more evidence for current clinical research.

## METHODS

This retrospective study included a total of 110 CHB patients who received treatment at The First People’s Hospital of Linping District, Hangzhou from January 2021 to January 2023. Clinical data of the patients were reviewed and the patients were classified according to the treatment received: TDF group (n=53, patients received TDF treatment) and ETV group (n=57, patients received ETV treatment).

### Inclusion & Exclusion Criteria:

Patients diagnosed with CHB (HBsAg positive ≥ 6 months), Child-Pugh classification at class A-B, aged over 18 years old, and with complete clinical data were included. Patients receiving immunosuppressive therapy or with history of immunodeficiency, with HIV infection, alcoholic liver disease, non-alcoholic fatty liver disease, hepatocellular carcinoma or any other malignancy, or other types of hepatitis viruses, and pregnant women were excluded. Patients treated with TDF or ETV less than 48 weeks were also excluded.

### Ethical Approval:

The Medical Ethics Committee of the First People’s Hospital of Linping District, Hangzhou City approved this study (No.: 2022075; Date: July 25, 2022).

### Treatment methods:

Patients in the TDF group orally took TDF (Hunan Qianjin Xiangjiang Pharmaceutical Co., Ltd.; Approval number: H20203590), 300mg/time, once a day. Patients in the ETV group orally took ETV (Haisco Pharmaceutical [Meishan] Co., Ltd.; Approval number: H20130031), 0.5mg/time, once a day.

### Collected information:

The outcome indicators were recorded after 48 weeks of treatment:

- ***HBV DNA levels*** were measured in the serum of 3ml of fasting elbow vein blood using fluorescence quantitative polymerase chain reaction assay kit.
- ***Liver function:*** The patient’s total bilirubin (TBIL), aspartate transaminase (AST), and ALT indicators were detected using the Roche fully automatic differentiation instrument Cobas 701.
- ***HBeAg seroconversion rate:*** Full quantitative detection of HBeAg was performed using the i2000 system.
- ***ALT normalization rate and HBV DNA conversion rate***: The normal ALT value was 5-40U/L. HBV NDA level >103/ml (as determined by polymerase chain reaction) was considered positive; ALT normalization rate was calculated as number of patients with normal ALT after the treatment/total number of patients; HBV DNA conversion rate was calculated as number of HBV DNA negative patients after the treatment/total number of patients.
- ***Adverse reactions:*** including vomiting, nausea, muscle pain, and stomach discomfort.

### Clinical efficacy evaluation:

The clinical efficacy was classified into three levels:

- Significant effect: clinical symptoms disappear, liver function returns to normal, and HBV DNA levels decrease by more than 45% from baseline;
- Effective: clinical symptoms improved, liver function improved, and HBV DNA levels decreased by 35% to 45% of the baseline value;
- Invalid: did not meet the above standards or progressed. Overall efficacy rate = (significant effect + effective)/total number of patients × 100%.

### Statistical analysis:

Data were analyzed using SPSS version 26.0 (IBM Corp, Armonk, NY, USA). Quantitative data were represented by mean ± standard deviation, independent sample t-test was used for inter group comparison, and paired t-test was used for intra group before and after comparison. Count data was analyzed using chi square test. *P*<0.05 indicated statistically significant difference.

## RESULTS

**Fig.1:**
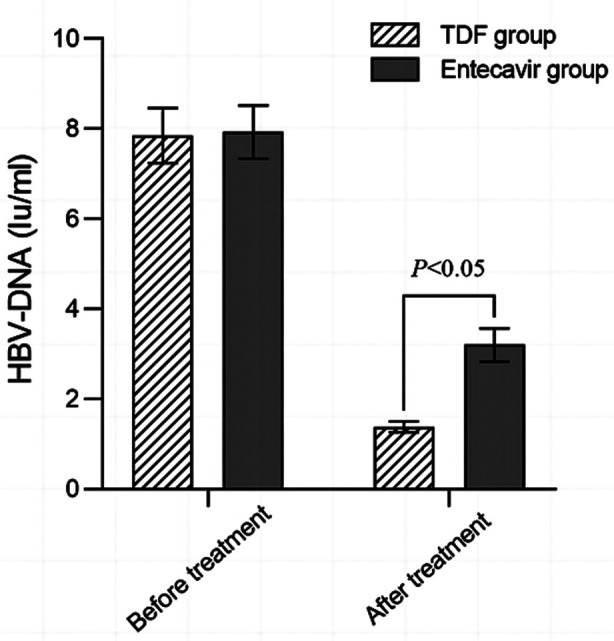
Comparison of HBV DNA levels between the two groups before and after treatment; Hepatitis-B virus deoxyribonucleic acid (HBV DNA).

A total of 110 patients met the eligibility criteria of this study, 53 cases in the TDF group, and 57 cases in the ETV group. There was no statistically significant difference in baseline data between the two groups of patients (*P*>0.05), Table-I. After treatment, the overall clinical efficacy in the TDF group (94.33%) was significantly higher than in the ETV group (78.95%, *P*<0.05), Table-II. Before treatment, there was no statistically significant difference in HBV DNA levels between the two groups (*P*>0.05). After treatment, HBV DNA levels decreased in both groups, and were significantly lower in the TDF group compared to the ETV group (*P*<0.05), Fig.1.

**Table-I:** Comparison of baseline data between the two groups.

Basic information	TDF group (n=53)	ETV group (n=57)	χ^2^/t	P
Gender (male/female)	29/24	34/23	0.273	0.601
Age (years)	47.81±8.45	46.72±10.80	0.593	0.555
Duration of disease (years)	2.52±0.99	2.38±0.94	0.751	0.454
Body mass index (kg/m²)	23.26±3.43	22.87±2.84	0.661	0.510
Family History (Yes)	20 (37.74)	23 (40.35)	0.079	0.779
Child Pugh grading of liver function			0.776	0.378
A	21 (39.62)	18 (31.58)		
B	32 (60.38)	39 (68.42)		

**Table-II:** Comparison of clinical efficacy between the two groups.

Group	Significant effect	Effective	Invalid	Overall efficacy
TDF group (n=53)	21 (39.62)	29 (54.72)	3 (5.66)	50 (94.34)
ETV group (n=57)	10 (17.54)	35 (61.40)	12 (21.05)	45 (78.95)
*χ^2^*	-	-	-	4.599
*P*	-	-	-	0.032

**Fig.2:**
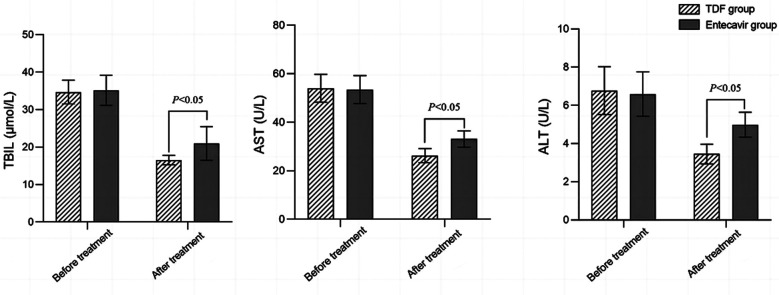
Comparison of liver function indexes before and after treatment between the two groups; total bilirubin (TBIL); aspartate transaminase (AST); alanine transaminase (ALT).

Before treatment, there was no statistically significant difference in the levels of TBIL, AST, and ALT liver function indicators between the two groups (*P*>0.05). After treatment, liver function indicators decreased in both groups, and were significantly lower in the TDF group compared to the ETV group (*P*<0.05), Fig.2. HBeAg seroconversion rate, ALT normalization rate, and HBV DNA negative conversion rate in the TDF group were significantly higher than those in the ETV group (*P*<0.05) Table-III. There was no significant difference in the incidence of adverse reactions between the two groups (*P*>0.05), Table-IV.

**Table-III:** Comparison of HBeAg seroconversion rate, ALT normalization rate and HBV DNA negative conversion rate between the two groups.

Group	HBeAg seroconversion rate	ALT normalization rate	HBV DNA negative conversion rate
TDF group (n=53)	40 (75.47)	43 (81.13)	44 (83.02)
ETV group (n=57)	31 (54.39)	33 (57.89)	35 (61.40)
*χ^2^*	5.336	6.944	6.340
*P*	0.021	0.008	0.012

***Note:*** Hepatitis-B e antigen (HBeAg); Hepatitis-B virus deoxyribonucleic acid (HBV DNA); alanine transaminase (ALT).

**Table-IV:** Comparison of incidence rates of adverse reactions between two group.

Group	Vomit	Nausea	Myalgia	Gastric discomfort	Total incidence rate (%)
TDF group (n=53)	2 (3.77)	1 (1.89)	1 (1.89)	1 (1.89)	5 (9.44)
ETV group (n=57)	1 (1.75)	1 (1.75)	1 (1.75)	0 (0)	3 (5.25)
*χ^2^_Yates_*					0.225
*P*					0.635

## DISCUSSION

This study found that TDF treatment was associated with overall higher total efficacy compared to ETV regimen, which is different from the findings by Sriprayoon et al.^10^ and Jeong et al.^11^ This difference in the efficacy may be due to the mechanism of TDF action. TDF is metabolized into the active form that binds to the reverse transcriptase of HBV, effectively inhibiting its activity and blocking viral DNA synthesis process.^7^-^9^,^14^ Continuous treatment can maintain low or lower levels of HBV DNA, reduce the risk of virus replication and drug resistance development, help control the course of the disease, and improve clinical efficacy.^14^-^16^ Our results show that HBV DNA levels in the TDF group are lower than those in the ETV group after the treatment. Our results are consistent with previous reports. A 2021 study by Lockman et al.^9^ showed that TDF is an effective antiretroviral therapy for HIV. Pan et al.^17^ also found that TDF has good antiviral ability in women of reproductive age with chronic HBV.

Studies show that HBV infection causes liver cell damage and inflammation, which prevents normal metabolism and excretion of bilirubin.^1^,^2^,^18^ In this study, levels of TBIL, AST, and ALT in the TDF group were lower than those in the ETV group after treatment, indicating better improvement in liver function in this group of patients. We may speculate that by inhibiting the synthesis of HBV DNA and subsequently, virus replication and proliferation, TDF reduces liver cell damage and the release of AST and ALT.^19^,^20^ Moreover, TDF can directly act on liver cells, protecting them from viral infection and damage. This helps to reduce liver cell necrosis and chronic inflammatory response, ultimately lowering the elevation of TBIL, AST, and ALT levels.^19^,^20^

The results of this study showed that the HBeAg and HBV DNA conversion rates, as well as ALT normalization rates, were higher in the TDF group compared to the ETV group. Positive HBeAg indicates active viral replication, while negative HBeAg indicates inhibition or disappearance of viral replication, which is an important indicator for the treatment of Hepatitis-B.^21^ TDF can inhibit excessive activation of the immune system, reduce inflammatory response, and help restore normal function of the host immune system. By regulating the immune response, TDF can promote the conversion of positive HBeAg to negative.^21^,^22^ We may speculate that TDF reduces virus replication and production by regulating immune responses. Specifically, when HBV invades the human body, it will trigger the immune response, in which natural killer cells (NK cells) will attack infected liver cells and trigger a release of cytokines, such as interferon, which can activate immune cells, and eventually lead to negative HBV DNA.^14^,^17^,^22^ Our results showed that both treatment regimens had similar low incidence of adverse reactions, indicating good treatment safety in both groups. Therefore, clinical choices can be made based on the actual situation of individual patients.

### Limitations:

Firstly, this is a single center retrospective study. Secondly, neither group was randomly assigned, and baseline information may be imbalanced and biased. Thirdly, a longer follow-up period is required to validate the results. Further higher quality research is needed to validate our results.

## CONCLUSION

Compared with ETV, TDF has comparable adverse reaction profile but has more significant clinical effects in patients with CHB, improving HBeAg seroconversion rate, ALT normalization rate, and HBV DNA negative conversion rate. TDF is associated with lower HBV DNA levels after treatment and better improvements in liver function of patients.

### Authors’ contributions:

**HW:** Conceived and designed the study. **HW** and **LW:** Collected the data and performed the analysis. **HW:** Was involved in the writing of the manuscript and is responsible for the integrity of the study. All authors have read and approved the final manuscript.
